# The Hospital. Nursing Section

**Published:** 1906-11-17

**Authors:** 


### The Hospital.

### Nursing Section.

Contributions for "THE HOSPITAL," should be addressed to the EDITOR, "THE HOSPITAL" NURSING SECTION, 28 & 29 Southampton Street, Strand, London, W.C.

NO 1,052.—VOL. XLI. SATURDAY, NOVEMBER 17, 1906.

### Notes on News from the Nursing World.

### OUR CLOTHING DISTRIBUTION.

THE matron of an East End hospital writes to remind us that upon a few occasions we have sent her a gift of clothing at Christmas, and to express the hope that we may be able to do so again this year. She adds, "I should be very grateful if you could, as I need scarcely say what a great help such a gift is, and how very much it is appreciated." This is but one of many of the appeals which we trust our readers, in the kindness of their hearts, will enable us to meet. We have to acknowledge the receipt of contributions from:—M. J. S.; A Private Nurse, St. Leonard's-on-Sea; and Mrs. Morley and Nurse Morley, Brockley. All parcels must be sent addressed to THE EDITOR, 28 and 29 Southampton Street, Strand, London, W.C., and should have "Clothing Distribution" written on the outside.

### PROMOTION IN THE ARMY NURSING SERVICE.

FURTHER questions respecting Army nurses were put by Mr. Rendall to the Secretary of State for War in the House of Commons on Thursday last week. In reply to one respecting the alphabetical arrangement of the names of members of Queen Alexandra's Imperial Military Nursing Service, Mr. Haldane stated that it was made at the request of the Nursing Board in December 1902. It was also, Mr. Haldane said, decided at that time that promotion in the service should be by selection and not by seniority. We believe that, on the whole, this principle has worked well. The system of promotion by seniority is naturally preferred by some of the members of the service, but promotion by selection is obviously more likely to ensure promotion according to merit.

### WHITECHAPEL INFIRMARY AND THE CENTRAL MIDWIVES BOARD.

THERE ought to be very good reasons for refusing an important nurse-training school the recognition of the Central Midwives Board. According to the statement of the matron of Whitechapel Poor-law Infirmary to our Commissioner, which will be found on another page, Dr. Wanklyn, after an exhaustive investigation, said that the results were excellent both as respects the cases and the success of the nurses at the examinations. Yet the Midwives Board has declined to recognise the institution on the ground of structural defect. This, however, has not been specified. The department is in charge of a sister who, like the matron and the assistant matron, holds the certificate of the Central Midwives Board. There is an average of 125 cases every year, and it might be considered that these advantages would override any ordinary structural deficiencies. Seeing that the number of qualified midwives is notoriously insufficient to meet the demand, it is a matter for regret that there should be any lack of encouragement to take the training on the part of the constituted authority. We do not wonder that the Local Government Board resent the exclusion of such institutions as the Whitechapel Infirmary, and desire accordingly to amend the rules of the Midwives Board.

### LOYALTY AND LAW.

IT seems rather ungracious for the Local Government Board to uphold the surcharge made by the District Auditor on the Chorlton Guardians. The surcharge was for a sum of money, not exceeding £80, which was spent on the occasion of the visit of the King and Queen to Manchester last year, chiefly in order that the nurses and children at their Cottage Homes might see the royal procession, which passed the offices of the Chorlton Union. The nurses, of course, enjoyed the sight exceedingly, and they certainly could not have witnessed it unless stands had been erected for the purpose. But in cases like these it would be far better for Guardians to seek legal advice before they decide to expend money for other than recognised purposes. If in this instance they had been assured that the expenditure would be contrary to law, friends of the nurses and children would probably have come to the rescue.

### MALE NURSE AND MEDICAL SUPERINTENDENT.

AN action brought against Dr. Neal, Medical Superintendent at Wandsworth Poor-law Infirmary to recover damages for an alleged slander by a male nurse, which was tried in the High Court of Justice last week, resulted in a verdict for the defendant. We do not think that the plaintiff was well advised in bringing the action, because in any case the words complained of were spoken by Dr. Neal on a privileged occasion. But it is unfortunate that the latter, having given the male nurse a testimonial setting forth his ability as an attendant in the mental and epileptic wards of the infirmary for eighteen months, should have subsequently—as it was fully admitted—confused him with a patient under treatment and denied all knowledge of him as a nurse.

### PROBATIONERS AND LECTURES.

AT the last meeting of the Belper Guardians it was reported that the superintendent nurse had undertaken to deliver lectures to the probationers on payment of £9 for forty afternoons' work, the probationers to pay half the total amount. It was added, however, that the probationers had decided not to take advantage of the lectures. Some of the Guardians protested against the decision. This is an astonishing position for an institution to take up, especially at Belper, where the certificate does not secure full qualification under the Poor Law.

### AN EXPERIMENT AT GOSPORT.

At the annual meeting of the Victoria Society for Nursing the Sick Poor in Gosport and Alverstoke it was unanimously decided to make a new departure. Last year the three district nurses paid 10,329 visits, but one of these was only partly engaged in the work. It has now been determined that for the next twelve months a part of the time of the third nurse shall be occupied in visiting persons who are willing to pay a small fee. The scale eventually fixed is 6s. to 12s. per week for one daily visit; 10s. to 20s. for two daily visits; 1s. to 2s. 6d. for a single visit; and 5s. to 15s. attendance at an operation. We have frequently deprecated the combination of district and private nursing under one management. But as the new arrangement at Gosport is only to be made as an experiment for twelve months, we do not blame the Society. The result, at any rate, should be to show whether there is, or is not, scope in the town and locality for the visiting nurses, whose failure to obtain adequate employment in the metropolis has lately been so conspicuously attested.

### GRANTS BY GUARDIANS.

At a recent meeting of the Bishop's Stortford Guardians it was decided to renew grants to all the local nursing associations which had applied and submitted their balance-sheets; but at a meeting of a neighbouring Board, the Saffron Walden Guardians, an application for a grant to a single nursing fund was refused. One of the Saffron Walden Guardians contended that if they subscribed to nursing associations private individuals would give up doing so. We must admit that there is some force in this argument; but the usual and reasonable ground on which Guardians are asked to subscribe to nursing organisations is that the district or parish nurse appreciably reduces the number of inmates of the workhouse infirmary or sick wards. When it is clear that this is the case, we feel sure that private subscribers will be all the more willing to do their part.

### PROBATIONERS AT DEVONPORT.

At a recent meeting of the Devonport Guardians Mr. Preston Thomas, Local Government Board Inspector, was present, and made some complimentary remarks respecting the training of probationers under their auspices. We note that he prefaced his compliments by remarking that, "after a great deal of trouble on the part of the medical officer and the superintendent nurse," a useful system had been established, and that he went on to speak of the difficulty of Poor-law institutions obtaining skilled nurses. This difficulty could be largely obviated if, as at Devonport, the recommendations of the medical officer and the superintendent nurse were more frequently adopted by Guardians.

### THE TRAINING OF MIDWIVES.

Princess Henry of Battenberg has consented to attend a meeting under the auspices of the Association for Promoting the Training and Supply of Midwives to be held in June next year. The object of the meeting is to give a fresh impetus to public interest in the work of the organisation. At a meeting of the Council last week a report, which was read by the Chairman of the Executive Committee, showed that, owing to lack of funds, the training of midwives by the Association had to be undertaken on a much smaller scale this year than last.

### THE MISSION FIELD IN SOUTH AFRICA.

According to a letter sent home by one of the Bishop of London's deaconesses, there is work to be done in the mission field in South Africa by trained nurses who are willing to give their services. The deaconess, who writes from Newland, East London, Cape Colony, states that a trained nurse is going out to help after Christmas, but she would be glad to hear of two or three more who would be willing to tend the sick. The country, she adds, is very beautiful, one of its attractions being that oranges, figs, grapes, and pomegranates, with other varieties of fruit, are to be had in profusion. It is possible that some of our readers may be willing and able to respond to the call.

### NURSING EXHIBITION AND CONFERENCE.

Full particulars have now been issued of the Nursing Exhibition and Conference on Nursing to be held next week. We have already given most of these, but it may be repeated that the Conference on Thursday is on the Care of the Consumptive, on Friday on Maternity Nursing, and on Saturday on Mental Nursing. The Conference, which, as well as the Exhibition, will take place at St. George's Hall, Mount Street, Berkeley Square, and commence daily at 8 P.M. Admision will be free. We are asked to state that any nurse having an invention she wishes to exhibit should communicate with the Hon. Secretary, Miss Barton, Chelsea Infirmary, S.W.

### REST AND RECREATION.

The opportunity is again kindly afforded by Mrs. C. S. Henry, of Parkwood, Henley-on-Thames, to trained nurses of enjoying rest and recreation, free of charge, in her beautiful Home. The invitation is for two weeks, but a longer or shorter period may be arranged as circumstances and the necessity of the case require. All applications should be addressed to the matron at the Home, who will be pleased to give any further information that may be desired. The Home is two miles from Wargrave Station.

### APPRECIATION OF TRAINED DISTRICT NURSES IN IRELAND.

A party of members of Parliament who lately visited the congested districts of Ireland were greatly impressed with the value of the work done by the fourteen nurses whose appointment is due to the efforts of Lady Dudley. They affirm the urgent need for its extension, which, however, cannot be secured without some further public support. In the recently issued report of the Viceregal Commission on Poor-law Reform in Ireland, reference is also made to the work, and its success is attributed mainly to the fact that none but highly qualified nurses of experience, aptitude and resource were selected. There seems reason to hope that the movement initiated in Ireland by Lady Dudley may thus not merely be for the benefit of the sick poor, but may also have the result of raising the general standard of nursing in the sister island.

### A SOMERSETSHIRE APPOINTMENT.

The Somerset County Council recently advertised for an inspectress of midwives, and amongst the conditions laid down it was specified that the person appointed should give her whole time to the duties of her office. There were 150 applicants, and we are informed that the two selected candidates were interviewed by four gentlemen and three ladies, who act as a sub-committee, the appointment being given to a nurse who recently accepted, and, it is said, intends to hold the position of superintendent of the Somersetshire Nursing Association.

### A NOVEL OBJECT-LESSON.

At the last of a series of lectures on Home Nursing given by Miss Abrahams, the superintendent of the Victoria District Nurses' Home, Huddersfield, the lecturer washed and dressed a life-size doll in order to illustrate the correct method of handling a newly born child. She afterwards, by request, gave a descriptive account of the work amongst the sick. The proceeds from the course of six lectures amounted to £13.

### A LOSS TO MACHYNLLETH.

Appropriate reference was made at the annual meeting of the Machynlleth and District Association to the death of the Dowager Marchioness of Londonderry. The Secretary said that the Association had lost an adviser and a friend; and Nurse Price, who subsequently thanked the meeting for their appreciation of her services, paid a warm tribute to the assistance rendered by the Dowager Marchioness, and attested her popularity among the people whom she herself was in the habit of visiting. The most practical way in which the latter can show their appreciation of the deceased lady is by more liberally supporting the organisation in which she was deeply interested. The Treasurer pleaded for subscriptions to the extent of £100 a year as a minimum, and in a prosperous town like Machynlleth, there ought not to be the slightest difficulty in collecting this sum.

### MATERNITY NURSES AND WOMEN'S SUFFRAGE.

On Saturday the first meeting of the Winter Session was held at the General Lying-in Hospital, York Road, Lambeth, when the question "Should Women Vote?" was answered in the affirmative in a paper read by Sister Olive, the midwife of the hospital. A paper on the other side was by Miss Victoria Hayden. This was followed by a number of three-minute papers contributed by the nurses. A Guy's sister was in the chair, and in summing up she pointed out that on the whole the feeling was in favour of deferring the granting of a vote to women until they were better qualified to exercise it. On the vote being put to the meeting, however, it was found that the majority were in favour of Women's Suffrage.

### A DEFICIT CONVERTED INTO A CREDIT BALANCE.

The feature of the report which was read at the annual meeting of the Dundee Sick Poor Nursing Society was that the financial statement showed that the debit balance last year of over £44 had been converted into a credit balance of £60. Several speakers warmly eulogised the work of the nurses, who last year attended 2,014 cases, and paid nearly 47,000 visits. The Rev. W. Lyall Wilson alluded to them as "ministering angels of healing"; and medical men said that their work was a help and supplement to that of the doctors. The Chairman intimated that it would be necessary to secure additional nurses in the near future.

### STARTING A NURSE AT TETBURY.

The authorities of the Cottage Hospital at Tetbury having decided that in future no nursing shall be done under their auspices outside the walls of the building, a movement has been set on foot for starting an independent district nurse. At a meeting held in the Town Hall it was urged that a fully qualified person should be engaged; but one of the speakers said that while a Queen's nurse would cost between £80 and £100 a year, one with not quite such good qualifications could be obtained for between £60 and £70. No decision on this point appears to have been arrived at; but in view of the statement of the Vicar of Tetbury that an income of £56 was already practically secured, and that he saw no difficulty in getting another £50 a year together if all classes would subscribe, we strongly advise the Committee of the new organisation to make the best possible start. The engagement of a Queen's nurse will, in the long run, tend to economy, because it will ensure efficiency, and will therefore gain the Association the confidence of the public.

### SHORT ITEMS.

Miss Davidson invites members of the Nurses' Union to her Nurses' Bible Readings at Friedenheim, Swiss Cottage, on Fridays, 5.15 to 6 p.m. Every Thursday evening at 26 George Street, Hanover Square, W., Miss Dashwood is at home. All nurses will receive a cordial welcome.—The Education Committee of the Hampshire County Council have resolved to make a grant of £60 from the higher education fund to be allocated to nursing scholarships.—Six candidates from the Chorlton Union Hospital who went in for the recent examination of the Central Midwives Board in Manchester were all successful.—An enjoyable evening was spent by the in-patients at the Cancer Hospital on Monday, when they were entertained by Miss Cara Bennett and her friends.

### The Nursing Outlook.

"From magnanimity, all fears above;
From nobler recompense, above applause,
Which owes to man's short outlook all its charm."

### WOMEN WORKERS AND HOSPITAL ABUSE.

Hospital abuse would soon be abolished if each hospital had available accurate knowledge of the means and circumstances of each applicant for relief. On a former occasion we have pointed out, that, if London was so organised as to have in each district an efficient and adequate system of district nurses, the hospitals might, with their aid, secure the prompt admission of all suitable cases from a medical or surgical aspect, and so immeasurably increase the value of the services which hospitals render to the suffering poor in a great city. Close and intimate relations between hospital authorities and district nurses would have the further advantage of enabling the former to have on their register full particulars of the social position of each patient admitted to treatment.

The Hospital Sunday Fund through its distribution committee has done its best to provide that every voluntary hospital shall have a special and efficient officer to detect abuse in the out-patient department. This action has given an impetus to the demand for suitable and trained women workers who are capable of filling this post with efficiency. Such an almoner's work is peculiarly adapted to the gifts which many women possess, for it not only requires training and the exercise of tact and discretion, but affords a wide sphere for the exhibition of genuine sympathy in dealing with many patients, who require counsel and advice, on the completion of their treatment at the hospital. We are induced to call attention to this matter, because we feel that many of our readers may desire to qualify themselves for the post of almoner, and at the present time there is an opening for several women of ability and education in this branch of hospital work. An application addressed to the secretary of the Charity Organisation Society at Denison House, Vauxhall Bridge Road, would, we have no doubt, elicit a reply containing information as to the conditions of training and the nature of the duties of this office. No almoner can in fact be efficient without previous training which, to prove effective, must include work with some local committee of the Charity Organisation Society, to be followed by experience in the work itself under an almoner at one of the hospitals. There is a growing desire to check hospital abuse and reduce the present amount of free medical relief at the voluntary hospitals, so that any woman who is well educated, possessed of some experience of life, in sympathy with the deserving poor, and imbued with the sound principle that all relief should be given with a view to increase and not to diminish the independence of the individual, may find a sphere of practical usefulness by qualifying as a hospital almoner.

The good which may be done by an efficient woman worker in this field becomes apparent when the work itself is described. There is nothing more fascinating probably than to become, by practice, a judge of character, for the more this experience is increased the greater is the influence for good which its possessor may exercise over her fellows. The almoner's first duty is to the hospital itself. It is essential to prevent any malingerer or mean person from receiving free medical relief, when an applicant is in fact unworthy of such consideration. A knowledge of the poor, their habits and ways, a keen insight into the reticence of many poor people with sensitive feelings, to whom poverty is a continual pain, a quick perception which detects the impostor, however plausible the story advanced, these are some of the necessary qualifications for a hospital almoner. In dealing with new cases each day it is essential to acquire the habit of rapid dispatch, thoroughness, and justice. It is a fatal thing for an almoner to spend so much time over one case that the rest have to be hurriedly and imperfectly disposed of.

Apart from their duty to the hospitals, the almoners have a duty to the patients. Here, by sympathy and the acquirement of a thorough knowledge of the various agencies and institutions which exist to render help to deserving cases, an almoner may immeasurably reduce the misery and suffering of a host of poor people whose lot would otherwise be unfortunate indeed. Very often when the surgeon has finished his work, the almoner can supplement it by securing, with promptness, the supply of a surgical appliance, or convalescent aid, whereby the patient is enabled to return to work, or to conserve and build up his strength, and so a whole family may be saved much misery and suffering. An educated woman must have her sympathies enlarged and her mind extended by acquiring a knowledge of the many charities with which an efficient almoner must be in close relation. The influence for good which she is enabled to exercise upon the poor people who learn to lean upon her in their hour of necessity, and the results of her influence in improving their character and benefiting those dependent upon them, is often considerable. Of course the remuneration of an almoner is not large, but the interest attaching to the work, and its importance to the poor, should attract some of the ablest of modern women to this occupation.

### Nursing in Tropical Climates.

By Andrew Duncan, M.D., B.S., M.R.C.P., F.R.C.S., Fellow King's College, Lecturer on Tropical Medicine at the London School of Tropical Medicine, and the Westminster Hospital.

### I.—THE NURSE.

The value of good nursing in disease is nowhere more apparent than in the tropics. My experience of the vast assistance on the one hand, and of the lack of help on the other, by the presence or absence of a reliable nurse has been gained by my service in India; but both these factors must be equally prominent in other parts of the tropical world. In India it often must happen that some days elapse before a nurse can be obtained for one's patient. During this time the amount of work and anxiety entailed on the physician in attending on the sick is not light, as he has in a bad case, in addition to his own duties, no rest at night. Hence, if anywhere, it is in the tropics that one has learnt to value the aid given him by the nurse.

I propose in these lectures to consider:—

A. The necessary qualifications of the nurse and the conditions necessary for preserving her health.

B. Her duties during the treatment of the several diseases.

C. Her duties as regards the preventive measures with reference to the diseases in question.

D. The precautions she should take as regards herself against disease.

### A.—The Necessary Qualifications of the Nurse and the Conditions Necessary for Preserving her Health.

1. *As regards Selection.*

(*a*) *Age.*—Owing to the time employed before the nurse is considered completely and fully educated in her profession, she attains to the age most suitable for embarking on her duties in tropical climates. Undoubtedly the period from 25 to 35 years of age constitutes the age during which she will best work in these regions. Before 25 she will be more liable to many of the diseases there prevalent; after 35 she will be too old to begin to work in the tropics. This fact is fully illustrated by the statistics obtained from the army in India. To take enteric fever, for instance. It is well known that this disease is most prevalent amongst soldiers of the age of 22 and under. Thus, taking the percentage of cases amongst the European troops in India during a certain year, we find the following:—

| Age | Per 1,000 of strength |
|---|---|
| 22 and under ... ... | 33.17 |
| 22 to 24 ... ... ... | 18.68 |
| 24 to 26 ... ... ... | 13.11 |
| 26 to 28 ... ... ... | 6.73 |
| 28 to 30 ... ... ... | 2.58 |
| Above 30 ... ... | 2.41 |

Colonel Welch, R.A.M.C., has shown that if men were excluded from India up to 25 years of age enteric fever would form but a very small proportion of the disease returns. During the Transvaal war, owing to the exigencies of the situation, there were only a few drafts from England; the time-expired men were retained, and thus the average age of the soldier serving in India was increased. What was the result? There was a drop in the admissions for enteric for the ages of those with under one year's service from 91.5 per 1,000 in 1898 to 30.4 per 1,000 in 1900. Again, the same thing is shown in the case of a second tropical disease, to wit, dysentery. Dr. Bryden here has shown that the admissions in the third year of service in India were one-half those of the first year. Lastly, it may be mentioned that the late Deputy-Surgeon Hewlett, C.I.E., recommended that soldiers should not be allowed to land in India until they had attained the age of twenty-five. It thus is seen that the nurse enters on her duties in the tropics at the right age.

(*b*) *Medical Examination.*—Nurses proceeding to the tropics are, or should be, medically examined. They, it is needless to state, must be thoroughly sound in all respects. There must, for instance, be no albuminuria, no asthma, no cardiac affections. With regard to a tendency towards phthisis, a few words may be said. It may happen that after qualifying in her education a nurse may show signs of a weak chest, and at the same time be desirous of continuing her vocation. Does a weak chest contraindicate a tropical life? According to my experience it does not; on the contrary, such a condition is benefited thereby. A friend of my own in 1880 showed evident signs of phthisis, and circumstances prevented his return to England. When I left India in 1900 his health was greatly benefited; although he had been exposed during his work to much labour, yet he had wonderfully improved. If he had been enabled to return to England it is doubtful if he would have been alive. My opinion on this question is shortly this. People with what is popularly termed a "weak chest" are not thereby debarred from serving in India, but they, as far as my experience teaches me, do much better there than in cold climates. Of course a nurse with actual disease should be rejected when in an incipient stage, not so much on account of herself, as on account of the patients she may have to attend upon. Hæmorrhoids should debar a person from going to India, as the affection will certainly increase there. Any liability to congestion of the liver should not be present.

(*c*) *Temperaments.*—The late Surgeon-General Sir William Moore, in his apposite remarks concerning temperament in its relation to the tropics, held that individuals characterised by a nervous temperament, marked by much energy, and a large amount of mental power, but existing in a frame, the chest of which is narrow, with languid circulation, pallid skin, and spare muscles, with movements now hasty, now languid, are not fitted for the tropics. They are prone to nervous maladies and congestion of the various internal organs. Their sensitive nature is ill-fitted to withstand the daily irritations that are the lot of those living in the tropics from the heat, the flies, the mosquitoes, the discordant notes of the "brain fever birds," the domestic worries of the servants, etc. The temperaments best fitted for the East are the so-called Bilious, or Bilio-sanguine, inasmuch as these are associated with the greatest endurance and the least sensibility to external impression. Next to these as regards suitability comes the sanguine temperament, inasmuch as there is a less liability to anæmia.

(*d*) *The regulation of one's life in the Tropics.*—(1) *Dress.*—One of the mistakes that individuals setting out for the Tropics commonly make is to take out a large outfit. In India everything can be obtained suitable for dress, and much cheaper than at home. One good suit should be taken for special occasions, and to serve as a pattern. Cotton drill, serge, etc., can all be obtained in the Presidency towns, or at the Elgin and Muir Mills, Cawnpore, or at the Basel Mission, Cannanore, whilst the native tailor, or dherzi, can copy anything—in fact, he does so too faithfully sometimes, as witnessed in the well-known story where the dherzi copied the patch of the suit that was given him. Linen or cotton should never be worn next the skin. These materials become soaked with perspiration, and need changing oft-times in the day. Now woollen material has great absorptive power of moisture, and is a great preservative against getting chilled: its power of permeability to air, so necessary for the healthy exchange of gases and a healthy atmosphere immediately round the body, is nearly double that of cotton. Again, the coolest things to wear in the hottest days in India are flannels. If you place two thermometers respectively under a blanket and a sheet during the day when the temperature, say, is 90° in the evening, the thermometer under the cotton will show much the higher range. An ample supply of boots and gloves should be taken, the former being kept off the ground or in a tin-lined box, and the latter wrapped up separately in tissue paper and kept in a large corked bottle or air-tight case to prevent them becoming mildewed. Cholera belts should always be worn: these can be best made out of a strip of flannel. Two pairs of neutral tinted glasses should be taken.

(*To be continued.*)

### The Nurses' Clinic.

### GUNSHOT WOUNDS AND SKULL INJURIES.

Although particular injuries do not always require special nursing, there are necessarily many points of difference, and the knowledge of these may spare a patient much pain and discomfort. The following injuries are not very generally met with, and therefore some hints as to the management of them may be useful to nurses who do not happen to have actually come across them during their training.

*Gunshot Wounds.*—These cases are not of very frequent occurrence in civil hospitals, but as so many nurses are taking up military nursing, and may some day be called upon for active service, they are included in the list. Though gunshot wounds are of three kinds, those made by bullet, explosive shell, or small shot, the nursing of all is practically the same. On the field, gunshot wounds are much more liable to suppuration as the first aid cannot be as thorough as at other times. The signs a nurse should give her attention to first, on admission of such a case, would be to those of hæmorrhage, shock, and pain. If hæmorrhage is very free, she should, if possible, stop it with the tourniquet, pending the arrival of the doctor. If internal hæmorrhage is present, she must lay the patient down with the head lower than the feet, by raising the foot of the bed, and applying ice to the injured area. For shock the patient would be wrapped in blankets, with hot-water bottles to the feet, a mustard leaf applied to the heart, and hot beef-tea, coffee, or brandy given internally. If hæmorrhage is present, brandy should not be given. Primary hæmorrhage is not general with these injuries, though it may occur. The temperature will be very low and the pulse feeble at first. If after about twenty-four hours or so, the temperature rises, the pulse quickens, the face becomes flushed, and the patient complains of headache, or becomes delirious, it points to suppuration having set in. Should this become very abundant septicæmia may follow through absorption of the discharge, and this will end in death. In favourable cases the temperature becomes normal in about ten days, the discharge lessens, and the wound granulates up. For the doctor the nurse must have plenty of disinfectant solution prepared, corrosive sublimate, or carbolic lotion, the hypodermic syringe with morphia to allay the pain, and ether or strychnine in case of collapse. Should there be doubt as to whether the bullet is still in the wound, antiseptic lotions, towels, bowls, etc., should be in readiness for the surgeon to explore the wound and remove the bullet. Immediately on admission the nurse should cover the wound with an antiseptic dressing, as the most important point in gunshot wounds is perfect asepsis. The subsequent nursing would be the same as that for wounds generally.

*Fracture of the Base of the Skull.*—In this injury much may be done by skilful nursing to help the patient. The symptoms are bleeding from the mouth or nose, or vomiting blood. The greatest danger is of septic meningitis setting in. The best means of guarding against this is to gently and thoroughly syringe the ears with warm solution of carbolic, place antiseptic gauze in the ear, and apply large pad of absorbent wool over the side of the head. The nose and throat should be well sprayed with alkaline antiseptic solution, if possible. The patient should be kept absolutely quiet, in a darkened room with an ice-bag to the head. The bowels should be freely opened, preferably with calomel, or some mercurial purge. Should it be a compound fracture of the base there will be a discharge of cerebro-spinal fluid through the nose and ears. Bed-sores must be carefully guarded against, the bed being kept dry and clean and free from wrinkles in the undersheet. All such cases must have mackintosh sheets and draw-sheets. The diet will be light and nourishing.

Cases of this kind must never be left alone for a minute, and much may be done by the nurse in arranging pillows and gently moving the patient very slightly from time to time to avoid continual pressure on the same parts, and by doing this she may save herself much future trouble by preventing bed-sores.

*Laceration of the Brain.*—The symptoms are fairly obvious. There may be a wound at the bottom of which the dura mater may be seen to be injured, and from which in some cases brain matter may be escaping. Frequently the patient is quite conscious. In most cases the bleeding escapes through the wound; if it is internal and enters the brain substance it is fatal. There may be no wound externally, nor even the appearance of a blow. As in "frac-

THE NURSES' CLINIC.—*Continued.*

tured base," the great danger is septic meningitis, therefore the wound must be rendered and kept aseptic. The hair should be shaved away from the wound, or better still, the head should be shaved, and rendered aseptic, as a thorough exploration will be made and probably trephining take place. If the dura mater is lacerated sutures and a gauze drain will most likely be put in. The temperature and pulse should be taken four hourly, and any sign of inflammation must be reported immediately. The room must be darkened and perfect quiet maintained. Any slight noise will upset the patient, who is generally in a very irritable condition. Squeaking shoes and rustling skirts should be especially avoided. Coals should be wrapped in paper and put on the fire, one piece at a time to avoid noise. Fluid diet only will be given for several days, small quantities at a time, and the bowels freely opened with calomel. If the patient is unconscious or only partly conscious, no efforts should be made to rouse him, except special orders to that effect have been given. While in an unconscious state the brain is more at rest than when the patient is conscious and able to think and worry.

*Fracture of the Nose* is always the effect of direct violence. When it takes place near the root of the nose it may be accompanied by injury to the brain. Should the bone be broken the nurse should prepare the patient, as well as she can, for an anæsthetic, as reducing the fracture is a very painful proceeding, and is rarely done without the administration of an anæsthetic. Strapping should be at hand as well as some strips of gauze soaked in carbolic acid, to plug the one side of the nose with after the bone has been replaced. Very often there is a good deal of bleeding which may be very tiresome to arrest. All simple means should be tried till the arrival of the surgeon—lay the patient down flat with the arms raised above the head, or put ice on the nose. Pinching the nostrils between thumb and finger will often prove successful, but if there is much swelling of the tissues and much pain this latter method could not be employed. In a case of fracture the nurse should never attempt to plug the nose, as she cannot know to what extent the nose may be injured. The nursing would consist in keeping the patient quiet, watching for hæmorrhage, and giving a light nourishing diet. The temperature and pulse should be watched for some days in case of any suppuration setting in.

### Incidents in a Nurse's Life.

### THE VALUE OF OBSERVATION.

The hour was 2 A.M. and the surgery empty and quiet. Presently a heavy step was heard approaching, and the night nurse went quickly forward to see what was required. A tired-looking man appeared, carrying a boy of eight years in his arms.

"'Ere nurse," he said, "my boy's got a bit of a cold, thought as 'ow doctor could give 'im somethink as can cure 'im, don't seem to get no rest of a night."

The child was suffering from something far more acute than an ordinary cold; this the practised eye of the nurse saw at a glance, and she lost no time in acquainting the doctor of the fact. He came at once in answer to the call. "Bad cold you say, father? It is worse than that, your boy has diphtheria and we must operate at once if you want us to save him." No sooner had the diagnosis been made than the tracheotomy instruments were on the spot and hastily sterilised. Meanwhile the patient, who was growing rapidly worse, had been removed to an examining room and oxygen given for momentary relief. His breathing grew still more difficult, and he was fast losing consciousness; an anæsthetic was quite unnecessary, and so with precision and skill the trachea was incised and the life-saving tube inserted.

Almost immediately the little sufferer began to revive, the cyanosis disappeared, and he sat up in a few moments and sweetly smiled upon his benefactors, little knowing how near he had been to death's door but a few minutes before.

Not more than five minutes, less time than it takes to tell the tale, had elapsed since his entrance to the surgery, and here he was practically restored to life and on the road to recovery.

So much for the skill of the surgeon and the nurse, whose training in both cases prepares them to meet any emergency and calmly do the right thing with the greatest possible speed.

Shortly after this the child was comfortably settled in the diphtheria ward and a special nurse in attendance. In a few weeks' time the little patient left the hospital bound for a convalescent home, where he is growing stronger each day and soon he will be at home again perfectly well, thanks to the hospital.

### The Nurses of Whitechapel Infirmary.

### INTERVIEW WITH THE MATRON. BY OUR COMMISSIONER.

It is due to the Whitechapel Guardians to say that the Infirmary under their control supplies an admirable example of making the best of available material. A new building is wanted, but in its absence everything appears to have been done that can be done to bring the existing one up to date. The nursing is necessarily carried on under difficulties which do not obtain in more modern palatial Poor-law institutions, but nothing struck me more on the occasion of my visit on a November afternoon than the air of cheerfulness pervading the commodious wards. Even the style of decoration, the predominance of green and red, contributes to this result; and as Miss Mowat, the matron, showed me round, I strongly felt the contrast between the appearance of comfort inside and the squalor of the adjacent streets. The fact that the patients wear red "nightingales" accentuates the brightness of the wards.

As we proceeded, the matron mentioned that the workhouse was converted into an infirmary in 1867, and that the original infirmary building is now utilised for overflow cases. That this accommodation is required may be judged by the statement that while provision is made for 590 patients this number is often greatly exceeded in the winter months.

"During last year," concluded the matron, "6,534 patients were admitted."

"And the full extent of your nursing staff?"

"There are 12 probationers, 25 staff nurses, five sisters, a night superintendent, an assistant matron, and myself."

"But are not most of the staff nurses still undergoing training?"

"Yes, most of them are in their second or third year, but some have passed their examinations and gained their certificate. We promote the probationers to be staff nurses in their second year as vacancies occur; and if the nurses decide to take up midwifery they have to remain a fourth year. Practically the training is for four years, but the fourth is not obligatory. The assistant matron, the night superintendent, and myself, all hold the certificate of the Central Midwives Board."

### The Midwifery Cases.

"And yet the Whitechapel Infirmary is not recognised as a school of training by the Central Midwives Board?"

"Unfortunately that is so, but beyond the plea of 'structural defect' no explanation has been given."

"You do not know what the structural defect is?"

"We know that Dr. Wanklyn, of the London County Council, who made a most exhaustive investigation, thought our results were excellent, both as regards the cases and the success of the nurses at the examination; but as we are now approaching the midwifery wards you can judge for yourself. The department consists, you see, of a room where the mothers are kept during the first stage, a labour room, and the general ward in which they recover."

"They all seem excellently arranged."

"I can assure you that we have a very satisfactory record, and, of course, the midwifery sister holds the certificate of the Midwives Board."

"How many cases do you have during the year?"

"An average of a hundred and twenty-five. Our nurses are allowed to qualify so far as the practical work is concerned, but they have to receive lectures outside from a practitioner who is recognised by the Midwives Board. We have hitherto encouraged our nurses to take midwifery training, but the refusal of the Midwives Board to recognise us interferes with the prestige of the infirmary and is disheartening."

### The Nurse-Training School.

"When did the infirmary become a nurse-training school?"

"This is one of the oldest schools under the Poor-law. A start was made when Dr. Larder came in 1887 as medical superintendent, with two probationers who had to go to a London hospital for lectures. In 1890 the probationers were increased to ten, with a trained nurse to teach them, and a regular course of lectures was then begun."

"How many were there when you came?"

"Ten. I added two, a midwifery sister, and three male nurses whom I did not include in my figures of the staff just now."

"You are yourself a hospital-trained nurse?"

"Yes, I was trained at St. Thomas's, but before my appointment here I was superintendent nurse at Birkenhead Poor-law Infirmary for a year; superintendent nurse at Shoreham Poor-law Infirmary for six years; and sister at Birmingham Infirmary for four years. As a rule, I think that Poor-law appointments should be given to nurses trained under the Poor-law."

"Is there anything special in the training here?"

"The house physician and surgeon give lectures on anatomy and physiology, and the medical superintendent takes up fever nursing and hygiene. I give a double course of lectures on practical nursing and bandaging; the assistant matron on sick cookery; and the night superintendent is a certified masseuse. She was trained here."

### How the Wards are Staffed.

"You choose your sisters from the staff nurses whenever you can?"

"We make a point of doing so because it is only fair. In addition to the departments which you have seen about 400 lunatics pass through the wards—two of 16 beds each—during the year. Qualified attendants, but no female trained nurses are employed. Generally speaking, the patients are treated on hospital lines, and our Board is one of the most considerate in London."

"How are your nurses distributed?"

"To each block of buildings, which contains beds varying in number from 80 to 60, a sister, a staff nurse, and a probationer are assigned to each ward during the day. At night there is a staff nurse in each ward, with the night superintendent in charge, and we have two relief nurses available. Some of the cases are exceptionally interesting, as the patients come from every quarter of the world and speak every language. We are really in the Ghetto."

### Salaries and Off-duty.

"Do you get plenty of applications for probationerships?"

"About 50 every year. The difficulty is to choose the best. The first year no salary is paid, but in the second £18, with a yearly rise to £30, £4 of this being in lieu of beer. I attach little importance to age; the great point is capacity. But we do not receive probationers younger than 21 nor older than 30. The probationers are off duty once a week from 2 to 9.30, and every day for two hours. Every third Sunday they are off from 2 to 9.30 p.m. The staff nurses get rather more off-duty, but all have a full hour for dinner, and all the staff but the first year probationers have three weeks' holiday. The night nurses get a night off once a month and two hours every day. I never refuse extra time, however, because the nurses here need recreation badly."

"I notice that they have a tennis-court."

"That is their only means of recreation on the spot. We have a tennis club, of which I am president. There are also good libraries."

### A Picturesque Arrangement.

The nurses' quarters are on the fifth floor, quite a climb. But they are very picturesque, and are arranged on the plan of a ship's saloon. Every nurse has a comfortably-furnished bedroom, with a fire-place, and there is separate sitting-room accommodation for probationers and staff nurses, with a piano and easy-chairs. The dormitories of the night staff are shut off by double doors, and the floors of the saloon being asphalted, the nurses can indulge in dancing without disturbing the patients.

Passing the up-to-date operating-room on our way down, and noting that each sister has a bed-sitting-room a little distance from the wards, I asked the matron about the diet.

"Here is to-day's bill of fare," she rejoined. "Breakfast, tea and coffee, kippers and haddock; dinner, boiled halibut and parsley sauce, roast beef, greens and potatoes, and milk pudding; tea, bread and butter, bananas; supper, fish, haricot mutton and cold beef. I am very careful about varying the diet. The night nurses have the same meals as the day, always freshly cooked. At midnight they get eggs and bacon. Three times a week coffee is provided for supper, the night nurses having cocoa."

"I think," concluded the matron, when I had congratulated her upon her own pleasant sitting-room, "that only a nice class of women would care to come here. They have to give up so much to live in Whitechapel, and, compared with nurses in different parts of London and the provinces, they have many difficulties to encounter. Happily, our medical men are a great help, and real enthusiasm is felt for the work."

### Central Midwives Board.

THE PENAL CASES.

A SPECIAL meeting of the Central Midwives Board, under the provisions of Procedure Rule 4, was held at Caxton House, Westminster, on Thursday of last week. Dr. Champneys presided, and there were also present Dr. Dakin, Mr. Parker Young, Mrs. Latter, Miss Wilson, and Miss Paget. The agenda contained a list of eleven penal cases.

The only midwife to answer the citation personally was Mary Ann Green, of Sutton, Surrey. She was charged with making a false notification of a still-birth to the local supervising authority and with contravening Rule E 15. Having heard the evidence and the defence, the Board decided that Mrs. Green should be severely reprimanded.

In the case of Cecily Dennett, Dr. Merry Smith, inspector of midwives for the city of Manchester, attended to give evidence in support of the charges, which alleged negligence in the case of five confinements. A letter from Dr. Buck, of Manchester, forwarded by Sir W. Sinclair, urged on Mrs. Dennett's behalf that she had brought up a family single-handed, was sober and clean, and had attended 300 cases during the year. It was decided that she should be severely reprimanded.

Mary Jane Wilson, of Workington, was ordered to be severely censured. It was complained that she neglected to supply herself with the appliances and antiseptics required by Rule E 2, and failed to keep a register of cases.

An infraction of the same rule and failure to send for a medical practitioner were alleged against Elizabeth Prior, of Wallingford. It was explained that Mrs. Prior was attending the case in question gratuitously. A request to be removed from the roll by the midwife herself was put before the Board, and, on the motion of Mr. Parker Young, it was decided to remove her name accordingly.

A similar course was taken with Fanny Simmonds, against whom charges were made of neglect in attendance on a case at Dallington. A communication was made to the Board that the midwife was suffering from a nervous breakdown, and was willing that her certificate should be cancelled.

Convictions in a court of law having been proved in the following instances, the four midwives concerned were struck off the roll :—

Betsy Simpkin, sentenced at Sheffield Quarter Sessions to three months' imprisonment for making a false registration certificate.

Ann Taylor, sentenced to three months' imprisonment in the second division at Liverpool Assizes for manslaughter.

Annie Twynam, sentenced at Clerkenwell Sessions for larceny.

Phoebe Ann Weaver, sentenced at Barnet Petty Sessions for altering a death certificate, and at Tottenham Petty Sessions for forging burial receipts.

No reply had been received from Esther Skeels, who was charged with neglecting to provide herself with appliances and with failing to keep a register, and it was decided to strike her name off the roll.

The same decision was arrived at in regard to Matilda Tee, of Portsmouth, against whom a series of allegations were made, in which want of cleanliness and the neglect of disinfectants figured. It was stated that the midwife was in her seventieth year, and could neither read nor write.

### Appointments.

[No charge is made for announcements under this head, and we are always glad to receive and publish appointments. The information, to insure accuracy, should be sent from the nurses themselves, and we cannot undertake to correct official announcements which may happen to be inaccurate. It is essential that in all cases the school of training should be given.]

ACCIDENT HOME, HEDNESFORD, STAFFORDSHIRE.—Miss Annie Blakemore has been appointed matron. She was trained at the Queen's Hospital, Birmingham, and has since been sister in the same institution.

BIDEFORD HOSPITAL.—Miss Evelyn A. Mansel has been appointed matron. She was trained at Charing Cross and Guy's Hospitals, London, and has since been matron of Kendal Hospital, and matron of the Children's Hospital, Bradford.

BRISTOL EYE HOSPITAL.—Miss Mary A. Harvey has been appointed sister. She was trained at the Bristol General Hospital, and was subsequently in charge of the two surgical wards, with the theatre, at the Jaffray Hospital, Erdington. She has also had experience in private nursing in England and abroad.

BURY INFIRMARY.—Miss Ada Scotson and Miss Jessie Dunning have been appointed sisters. Miss Scotson was trained at the Royal Albert Edward Infirmary and Dispensary, Wigan. She has since done sister's holiday duty in the same institution; has been senior charge nurse at Wrexham Infirmary, and has done temporary matron's duties. Miss Dunning was trained at the Cottage Hospital, Warminster, and the General Infirmary, Chester. She has since taken sister's holiday duty, theatre sister's duty, and night superintendent's duty, and had charge of the out-patients' department in the latter institution.

CHEPSTOW UNION.—Miss Gertrude Davis has been appointed head nurse. She was trained at Battersea and Clapham Maternity Hospital, and has been charge nurse at Shaftesbury Workhouse Infirmary.

HAMPSHIRE NURSING ASSOCIATION.—Miss Julie A. Jacobs has been appointed assistant to the superintendent of the Hampshire Nursing Association, affiliated to Queen Victoria's Jubilee Institute for Nurses. She received her district training at Hammersmith, and holds the Central Midwives Board certificate. She has worked as a Queen's Nurse at Alderley Edge.

KETTERING DISTRICT NURSING ASSOCIATION.—Miss Susannah H. Taylor has been appointed senior nurse of the Kettering District Nursing Association, affiliated to the Queen's Institute. She was appointed a Queen's Nurse in January 1905, and has since worked on the staff of the Camberwell Home.

KETTERING JOINT ISOLATION HOSPITAL.—Miss Eva McIlroy has been appointed nurse matron. She was trained at Wolverhampton and Shepperton General Hospital. She has since been charge nurse and assistant matron at Southampton Isolation Hospital, and matron of High Wycombe Borough Infection Hospital.

PLAISTOW HOSPITAL.—Miss Beatrice Scott has been appointed assistant matron. She was trained at the Croydon General Hospital, and has done private nursing. She has also been charge nurse at Tolworth Hospital, Surbiton, and ward sister at Plaistow Hospital.

ROYAL CORNWALL INFIRMARY, TRURO.—Miss Bessie Cuaff has been appointed matron. She was trained at the South Devon and East Cornwall Hospital, Plymouth, and has since been staff nurse and night sister at the Hospital for Women, Soho Square, London, and sister of the Female Landing at the Royal Cornwall Infirmary. Miss Eva E.

Jones has been appointed sister of the Female Landing. She was trained, and has since done holiday sister's duty, at the Royal Free Hospital, Gray's Inn Road, London.

Sanitary Hospital, Boscombe, Bournemouth.—Miss Maud Mellor has been appointed staff nurse. She was trained at the Victoria Hospital, Blackpool, and has since been casualty and assistant nurse of the light department at the East London Hospital for Children, Shadwell, E.

St. Mary (Islington) Infirmary.—Miss Mary Neile has been appointed sister. She was trained at Sir Patrick Dun's Hospital, and the Rotunda Hospital, Dublin. She has since been nurse at the London Throat Hospital, and charge nurse at North Dublin Infirmary.

Somerset County Nursing Association.—Miss Cathlin C. du Sautoy has been appointed superintendent of the Somerset County Nursing Association, affiliated to the Queen Victoria's Jubilee Institute for Nurses, and inspector of midwives under the Somerset County Council. She received her hospital training at Guy's Hospital and her district training at the Metropolitan Nursing Association, Bloomsbury Square, London. She holds the Central Midwives Board certificate. Miss Emma A. S. Pilgrim has been appointed assistant to the superintendent. She has worked on the staff of the Newhall Street Home, Birmingham, and subsequently at Kenilworth. She holds the Central Midwives Board certificate.

Victoria Infirmary, Northwich.—Miss Louisa Strickland has been appointed matron. She was trained at University College Hospital, London, and has since been sister in the same. institution, night superintendent at the Samaritan Free Hospital, London, and temporary theatre sister in the same institution. There were 111 applications for the appointment.

Walsall District Hospital.—Miss A. E. Macdougall has been appointed sister. She was trained at the Royal Infirmary, Preston, where she was afterwards appointed charge nurse of the operating theatre. She has since been staff nurse at the East London Hospital, where she also did temporary sister's duty. Previous to her training she was for six months at the Isolation Hospital, Colchester.

### Everybody's Opinion.

[Correspondence on all subjects is invited, but we cannot in any way be responsible for the opinions expressed by our correspondents. No communication can be entertained if the name and address of the correspondent are not given as a guarantee of good faith, but not necessarily for publication. All correspondents should write on one side of the paper only.]

### THE PENSION FUND AND CASES OF SICKNESS.

"Thankful" writes: I venture to ask for space in your valuable paper as a means to bring before my sister nurses the benefits of the Royal National Pension Fund for Nurses in cases of sickness, in addition to the advantages as a pension fund. As to the latter, I do not think I need say much, because it is admitted by all that there is nothing better than a provision for old age; and when one knows that one has nothing to fear, from a pecuniary point of view, with the approach of old age, it certainly relieves one of much anxiety. With regard to the sick allowance in connection with the Fund I cannot speak too highly. I have been incapacitated for sixteen weeks, and during the whole time I have had an allowance of 10s. per week, and this amount has been very useful to meet the many small extras which are indispensable in time of sickness. There has been no difficulty in connection with obtaining the sick allowance; merely a letter asking for it accompanied by a doctor's certificate. It is not until one is sick and receives the benefits that the advantage of being able to join such a Fund is realised, and it is with this sense of thankfulness that I appeal to you to insert this in your columns. If a nurse is fortunate enough not to require the sick allowance, she has the pleasure of knowing that her contributions are assisting others, and in addition to this there is always the knowledge that the aim of the contributor is to provide a "stocking leg" for the time when she can no longer perform efficiently the duties of her profession.

### THE STANDARD OF NURSING.

"M. G. B." writes: "Sister's" letter under the above heading deals with the social status of the nurse, and has nothing to do with the standard of nursing. The letter appears to be based on the common fallacy that all nurses are ladies; if not, they become so by becoming trained nurses. Therefore it is an insult to trained nurses to advertise for a nurse whose husband is a porter. Surely "Sister" must know that in the nursing profession all social grades are represented, and that women from all classes, from the duke's daughter to the dustman's, are to be found in different capacities in its ranks, and there is room and work for all. As there is no legal definition at present of a "trained nurse," the term is a very elastic one, and may mean anything or nothing. It is certainly a novel idea that a nurse is treated with a varying amount of respect according to her social standing. It is what a nurse is personally, not who she may be socially, that is her title to respect. It could hardly advance the standard of nursing to eliminate all who were not of gentle birth; the profession would be the poorer for the loss of a large number of first-rate nurses, and the number of ladies by birth available would by no means meet the nursing needs of the Empire. The standard of nursing, were such a class distinction made, would inevitably deteriorate. The gift of what, for want of a better term, I must call "nursing power" is not confined to any one class; material for training is to be found in all ranks of life, and to limit the field of choice for the nursing profession to a comparatively small class would certainly not be the way to ensure efficiency. "Nursing" has so many branches, and is carried on under such varied conditions, that nurses from all ranks of life are wanted in the profession. It is no more "an insult to trained nurses" to advertise for one whose social status is that of the porter than it is an insult to nurses in a humbler rank of life for the War Office to require Army nurses to be ladies. As to how a porter's wife would expect to be treated, may I remind "Sister" of the Frenchman who said that "he treated all men with courtesy and consideration, not because they were gentlemen, but because *he* was one."

### A STRANGE DISMISSAL.

A District Nurse writes: I wonder whether any other district nurse has been put in the peculiar position I have been. I am given a month's notice by my committee, which is composed of twenty-eight well-known ladies in the town, for not living in a home. I became district nurse seven years ago. When I had been here two years a second nurse was appointed. At the end of four years more, two being found not to be required, we both left, and a new nurse was appointed. She brought a maid with her, and a home was found for her, furnished by the committee, who had previously given the furniture to a home where the two nurses lived. As the new nurse left at the end of eight months and I was still in the town taking private cases, I was asked by some of the ladies to re-apply, which I was only too glad to do, and was appointed by nineteen votes to four. After giving up the district work I had taken rooms with a friend, a schoolmistress, and told the committee I could not manage a house, and for refusing to do so I am dismissed. The home contains sitting-room, front and back kitchen, committee-room, two bedrooms, and bathroom. The salary is £70 and furnished house. I told the committee I could not pay board and wages for a servant, nor had I time to superintend her work, and I asked to be allowed to remain with my friend. It is a large, rambling district, which takes me out usually from 8.30 a.m. to 1 p.m. and from 6 to 9 p.m. The secretary assures me that there is no fault to find with myself or my work. Am

I justified in feeling injured? Of course I have transgressed in not doing as my committee request. I was a Queen's nurse for five and a half years, but after my return to district work my committee preferred that I should not wear my badge, as they object to the visits of the inspector.

[We think our correspondent would be more comfortable if she resumed work elsewhere as a Queen's nurse.—Ed. The Hospital.]

### SO-CALLED NURSES' ASSOCIATIONS.

"A Trained Nurse" writes: I thank you for publishing my letter last week, and for taking sufficient interest in the same to append a "note," but I am afraid you have mistaken my meaning. The nurse I mentioned did not wish to join a permanent co-operation, but to get a few private cases or "temporary work" while waiting for a hospital post. What I specially complained of was the title "Trained Nurses' Association." When the office was full of domestic servants, why should the two be combined and called by that misleading title? My second complaint was the high fees charged for the salary given. Why should people be allowed to grind the less fortunate nurses down when they have to give three or (as in the instance mentioned) four of the best years of their life, at risk of health and very little salary, before they can be called "trained nurses"? "You say "the nurse mentioned appears to have accepted the conditions offered." Yes, in the absence of an alternative, with pretty low funds after a year's ill-health, being too proud to appeal to charity. And there are many others like her. But in the centre of all things and the "capital" of the world, why should it be necessary? I may add that the private institution who employed the nurse in the instance mentioned could well afford to pay the full fee.

### THE BREACH OF CONTRACT CASE.

Miss Heatley writes from St. Clement's Maternity Home and Nursing Institution, Fulham, : I shall be obliged if you will insert my reply to Mrs. Jukes's letter in your issue of last week. Mrs. Jukes asks, first, "What was the contract between Miss Heatley and myself?" Reply, Three months' training in midwifery, as her receipt shows. But three months' training in midwifery to untrained pupils (*i.e.* those who have had less than one year in a hospital) does *not* mean preparation for the C.M.B. examination. Pupils who enter to be prepared for that examination have their receipts worded "Three months' training in midwifery and preparation for C.M.B. exam." The latter part was not on Mrs. Jukes's receipt, and therefore showed that when I gave that receipt she knew (as I had pointed out to her) that she was not entitled to the C.M.B. training requirements. She then promised to pay the balance of the fees the first week in September. I have often given receipts in the same form as hers when pupils have paid by instalments. Judging from the number of letters I have received expressing indignation at the verdict of the judge, I think I may safely say that I did not commit "a breach of contract," and her own book shows that she was present at fifteen deliveries and acted as midwife at seven cases during the one month she was here. I have had no occasion to go to law before, therefore I was not up in such small points as "on account," so that I am afraid Mrs. Jukes has the advantage of me there. I would like to take this opportunity of thanking my numerous correspondents for their expressions of sympathy and loyalty.

A Midwife writes: I wish to make a few remarks in reference to the breach of contract case. The receipt of £16 16s. for three months' training in midwifery given by Miss Heatley to Nurse Jukes entitled her to three months' training in midwifery, which she was receiving. It was no receipt for midwifery training for C.M.B. Miss Heatley always adds "preparation for C.M.B. examination" if the pupils are being prepared for that examination, otherwise it is simply for the home certificate. The judge evidently considered that training in midwifery meant training for the C.M.B. examination, which it does not; and as Nurse Jukes was receiving midwifery training for which she held the receipt, Miss Heatley was not breaking her contract with her, having told her that training for the C.M.B. examination could not be done under four months by an untrained nurse.

"A Lover of Justice" writes: I was a pupil midwife at St. Clement's, and received my receipt for my fees at the same time as Mrs. Jukes. I am a trained nurse, and went to St. Clement's for three months' training for C.M.B. exam. Mine was as follows:—"Received £16 16s. for three months' training in midwifery, with preparation for the C.M.B." Mrs. Jukes' was as follows:—"Received from Nurse Jukes the sum of £16 16s. for three months' training in midwifery." No mention being made about preparation for the C.M.B.

### A LESSON FOR A DISTRICT NURSE.

"J. B." writes: For the past few days we have had nothing but rain and darkness in Edinburgh, making unsavoury places and people decidedly worse. It has been an effort to me to visit in the district. The other morning looked a little brighter and I felt very thankful and started on the daily round of visits, getting to Arthur Street, and climbing to the top flat of a long and dirty stair, to see a little lad twelve years of age suffering from abscesses, generally visited every other day. Entering the room with a "Good morning," the kind face looked up smiling and he said: "Nurse, I was glad that yesterday was not your day for coming to see me." When asked why, the little lad replied, "Because, nurse, it was so wet. It would save you." The little boy evidently thought that he might be my only patient. But was this not a lesson for me not to grumble or grudge visiting in the district any time? Even in the midst of poverty and crime the kindest thoughts are often found.

### Novelties for Nurses.

**(By our Shopping Correspondent.)**

### HOSPITAL CONTRACTORS AND NURSES' OUTFITTING ASSOCIATION, STOCKPORT.

In these days nurses need not complain that their wants are insufficiently catered for. At Stockport is an establishment which devotes itself almost exclusively to nurses' outfitting. The cloaks of the Hospital Contractors and Nurses' Outfitting Association are all made to measure, in very good quality cloth or serge, at most reasonable prices. The "Sister Mary" cloak is a particularly stylish-looking garment, made of shrunk navy cloth, faced with merve silk and a hood lined with the same. A detachable cape, also faced with merve silk, fastens in front with neat straps, which give the cloak a very smart appearance. The price, with hood, cape, etc., and faced with silk, is 27s. 6d.; with cape, but without hood, 25s.; without hood, cape, or silk facing, 22s. 6d. "The Muriel" cloak, circular with a round yoke, can be had from 22s. in cloth, or in all-wool serge, 25s. These cloaks are all tailor-made in the workrooms of the association, and can be made and despatched, if necessary, within twenty-four hours of receiving an order. Measurement forms are supplied, by the use of which a perfect fit can be relied upon. Aprons are also made to order, and can be despatched by return. "The Marvel" apron of linen-faced cloth, at 1s. 11½d., and "The Netley" linen apron, at 2s. 9d., are very favourable specimens, both as regards shape and the quality of material. Measurement forms are also supplied for aprons. "Sister Dora" caps, made with a drawing string, can be had plain at 6¾d. and hemstitched at 8¾d.; the "New Sister Dora," a very natty little cap, costs 7¾*d.* in fine nainsook, and in lawn 8¾*d.* Dainty strings of varying degrees of ornamentation can be had tucked at 4½d. a pair, hemstitched at 7½d. a pair, and fancy at 1s. 0½d. a pair. Belts (made in Ireland), possessing two sets of stud holes, are 10¾d. each. Bonnets, all trimmed with silk velvet, can be purchased at 5s. 11½d. each, or in fine pedal straw at 7s. 11½d; "The Gladys," at these prices, seems a particularly comfortable shape, and very light withal. "The Sister Helen" bonnet, with its brim turned straight back with velvet, is a very popular style. Veils for these bonnets cost 2s. 6d. extra.

### Notes and Queries.

**REGULATIONS.**

**The Editor is always willing to answer in his column, without any fee, all reasonable questions, as soon as possible.**

**But the following rules must be carefully observed.**

1. **Every communication must be accompanied by the name and address of the writer.**
2. **The question must always bear upon nursing, directly or indirectly.**

**If an answer is required by letter a fee of half-a-crown must be enclosed with the note containing the inquiry.**

*Sick Benefit.*

(82) Is there any sick benefit club or society for women, or could I join the sick benefit section of the Royal National Pension Fund without entering for a pension?—*Veto.*

There is no special sick insurance benefit of such a nature for nurses apart from the Pension Fund, in which you must be a policy-holder to be entitled to sick insurance.

*Lady Nurses.*

(83) Can you tell me where (1) lady nurses for children are trained, and (2) where a baby might be received for 10s. per week?—*Beta.*

(1) See answer to *Winsley*. (2) See answer to *Chiswick* (88).

Can you tell me any place where a young girl between 16 and 17 could be trained as a children's nurse? What is the length and expense of training?—*Winsley.*

The Norland Institute, 10 Pembridge Street, W.; the Liverpool Ladies' Sanitary Association, 8 Sandon Terrace; the Gloucestershire School of Domestic Service, Belmont House, Cheltenham; Princess Christian's Training College, 19 Winstow Road, Withington, Manchester.

*Massage Electricity.*

(84) What is the cost of instruction in massage and electricity, where can I train, and what is the fee?—*York.*

The fees vary from £10 to £40, and the duration from two weeks to three months. Write to the Incorporated Society of Trained Masseuses, 12 Buckingham Street, Strand, W.C., for advice. Have you inquired for a school of massage in Manchester?

*Nurses' Co-operations.*

(85) Please tell me of a good co-operation, London, where the age limit is above 30.—*Unsettled.*

The Nurses' Co-operation, 8 New Cavendish Street, receives nurses up to 35.

*Age of Probationer.*

(86) What is the earliest age at which a London children's hospital would receive a probationer? And would a general hospital take one at 20?—*Probationer.*

The Alexandra Hospital for Hip Diseases receives probationers at 20, but no general hospital until 23.

*Claim for Salary.*

(87) I have been engaged at a salary of £30 yearly, paid monthly. If my patient died a week or two after my engagement, could I claim a month's salary, or only for the week or day I had served?—*Eva.*

You would be entitled to one month's salary, or you might be asked to stay till the end of the month, whichever your employer preferred. In this case you could refuse to do what you were not engaged to do. An agreement should always be entered into before accepting a post. It need only be in the form of a letter from your employer, stating the salary, duties, term of notice, and privileges attached to the post.

*Motherless Baby.*

(88) Can you tell me of a home for a motherless baby four months old? A small weekly payment would be possible?—*Chiswick.*

Inquire of the Sesame Home, Acacia Road, St. John's Wood, N.W., or the Orphanage, 28 Upper Tooting Road, S.W.

*Open-air Shelter.*

(89) A woman suffering with the early stages of consumption desires to secure an outdoor shelter that she may pursue the cure. Could any of your readers help in the matter?—*Waltham Abbey.*

We cannot think it wise that the patient you mention should regulate her own cure. Could not the doctor you speak of or some influential person help to get admission for her into a consumption sanatorium, where she will have the good food and attention she requires? If you care to have it, we can send you a list of sanatoria with conditions of entrance.

*Pension or Assistance.*

(90) Can you suggest an institution which would receive a penniless young man suffering from a peculiar form of chorea, incapable of controlling muscular and nervous movements?—*North Kensington.*

Possibly the Sisterhood of St. John the Baptist, Clewer, might help you. Write to the Sister Superior, or one of the hospitals for incurables might be suitable.

*Holt Ockley Nursing Association.*

(91) Can you tell me where to get information about the Holt Ockley system of nursing? Are there any vacancies for probationers?—*H. T.*

Write to the Secretary of the Cottage Benefit Nursing Association. The address is Denison House, Vauxhall Bridge Road, S.W.

*Book Wanted.*

(92) Some time ago I saw a book on urine testing advertised. I think it was by Robinson, price 1s. Will you kindly help me to find the book?—*Inquirer.*

You probably mean "Urine Testing," by J. K. Watson. You can obtain this from the Scientific Press, 28 Southampton Street, Strand, for 1s. 1d., post free.

*Boils.*

(93) Can you tell me the reason of boils appearing in various parts of the body, in summer, and what is a simple remedy for diarrhœa?—*Rose.*

Both cases you mention should be referred to a medical man.

*Midwives.*

(94) Is there an association for midwives?—*Dragon.*

Yes. The Midwives' Institute, 12 Buckingham Street, Strand.

*Male Nursing.*

(95) (1) Will you tell me why male nursing has not progressed as it ought to have done? (2) Is it wise to give it up and go into the field again, or try something else? (3) Do you know of any other training schools giving certificates except the National Hospital and the Army hospitals?—*Derby.*

(1) Since you ask us, we must own the painful truth that, speaking generally, male nurses are not a success, because in many cases the best men do not take up nursing. They were tried in an American hospital, and it was found expedient to replace them by female nurses. (2) Would it not be better to continue an occupation you are familiar with, rather than experiment? (3) There are no other training schools for male nurses which give certificates.

*Age of Training.*

(96) Is there a good hospital either in London or in the provinces which receives probationers at 19?—*Rena.*

No general hospital receives probationers so young. You can learn all particulars of training schools in "How to Become a Nurse," London Scientific Press, 28 Southampton Street, Strand, W.C., price 2s. 4d. post free.

*Water-bed.*

(97) Which is the best way to preserve a water-bed when not in use?—*B. B.*

Partly filling for some time with warm water, say, about once in three weeks, is the only certain method of preserving it. But if you are not likely to use it, you had better sell and buy again.

*Central Midwives Board.*

(98) Should I be eligible for the Central Midwives Board examination, as I have not trained at one of the recognised schools? And, if not, could I if I had instruction from a recognised teacher?—*Homerton.*

Write to the Secretary of the Board, Caxton House, Westminster, S.W., and make your own inquiries.

*Visiting Nursing.*

(99) I am anxious for daily employment, say to 5 P.M. Is there any agency that will help me, or can you advise?—*Hopeful.*

Write to the Marylebone Daily Visiting Nurses' Association. The Hon. Secretary, 126 Seymour Place, W., and you might get advice.

*Male Nursing.*

(100) Can you tell me where a male nurse would get the best all-round training in nursing?—*Inquirer.*

You can get two years' training in the National Hospital for the Paralysed and Epileptic, Queen Square, Bloomsbury; and also the Army hospitals train orderlies, and these have experience in all kinds of nursing.

**Handbooks for Nurses.**

| | Post Free. |
|---|---|
| "A Handbook for Nurses." (Dr. J. K. Watson) ... | 5s. 4d. |
| "Nurses' Pronouncing Dictionary of Medical Terms" | 2s. 0d. |
| "Art of Massage." (Creighton Hale) ... ... ... | 6s. 0d. |
| "Surgical Bandaging and Dressings." (Johnson Smith.) ... ... ... ... ... ... ... | 2s. 0d. |
| "Hints on Tropical Fevers." (Sister Pollard.) ... | 1s. 8d. |

Of all booksellers or of The Scientific Press, Limited, 28 & 29 Southampton Street, Strand, London, W.C.

### Death in our Ranks.

There passed away suddenly, as the result of cerebral hæmorrhage, at the Home for Gentlewomen in Harley Street, London, last month, at the age of forty-four, one who for twenty-five years was intimately and actively connected with the nursing profession. Sprung from an old Herefordshire family, the late Miss Agnes W. Britten, on the death of her mother, decided to train as a nurse, more for the love of the work than as a means of livelihood. At the early age of nineteen she commenced her training at the Children's Hospital, Glasgow, and completed her course at the Royal Southern Hospital, Liverpool. Her first appointment was at the Wolverhampton and Staffordshire General Hospital, where for two years she was charge nurse of the male surgical ward and operating theatre. She was afterward for a short time sub-matron of the Home for Crippled Children at Clifton. On leaving Clifton she entered on a six months' course at the Rotunda Hospital, Dublin, receiving the qualifying certificate at the end of her term. Her next post was assistant matron at Kensington Poor-law Infirmary, where a year later she was appointed matron to a supplemental infirmary in connection with the same institution. In August 1893 she was selected out of a large number of applicants as matron of the Maternity Hospital, Glasgow, where for four years she so faithfully and well discharged her duties that on resigning the appointment on account of her health the director and hon. treasurer in a testimonial stated that, "without any disparagement to her able predecessors, the hospital had never had a more efficient matron." After an interval of rest, during which she became proficient in massage, she volunteered and was accepted for service in the Boer war, and at the end of the war received two medals and a clasp. Subsequently she settled at Johannesburg, where she established a nursing home. This did not prove a financial success, and her health failed. She was overtaken by an apoplectic seizure, which resulted in left hemiplegia and some disturbance of her sight. She was very anxious to get back to England, and after partial recovery returned to London last September. She entered the home in Harley Street on October 2 for the purpose of treatment, and thought herself improved up to the morning of her death, when she suddenly became unconscious and died the same afternoon.

### Wants and Workers.

### TO DISTRICT NURSES.

Lady Lyttleton, 12 Manchester Square, W., writes to recommend an elderly woman who would be glad in return for her food and a small weekly remuneration to look after an invalid under the direction of a district nurse. The neighbourhood of Hammersmith is preferred, but not essential. The person recommended is not strong enough for hard work, but would be intelligent, kind, and obedient to directions. Anyone knowing of such an opening is requested to send particulars to "M.B.," 30 Bridge Avenue, Hammersmith, W.

### A VERY DESERVING CASE.

Will someone give a mackintosh sheet and any old sheets and blankets to a very deserving case? A poor woman of eighty-four has been bedridden for eighteen years with rheumatism. She lately fell out of bed and fractured her ankle. Her only supporter is a blind daughter, who earns a scanty living, partly by knitting socks, cuffs, and scarves, and partly by alms given her. This poor woman has also got tubercular disease in one leg and has had epileptic fits. They are looked after by a granddaughter of the former, a girl who is now only twenty, and who has been doing this ever since a small child. She does her best to keep them clean, but without the necessaries this is most difficult. Any old garments for the daughter, who sits out in an old invalid chair in all weathers, and a cast-off rug would be most acceptable, and will be gratefully acknowledged by Nurse Agnes, 63 Shanklin Road, Brighton.

### For Reading to the Sick.

### HEAVENWARD, HOMEWARD.

And some with eager longing go, still pressing forward, hand in hand,
And some with weary step and slow, look back where their Belovéd stand—
Yet up the misty stair they climb, led onward by the Angel Time.

As unseen hands roll back the doors, the light that floods the very air
Is but the shadow from within, of the great glory hidden there—
And morn and eve, and soon and late, the shadows pass within the gate.

Complain not that the way is long. What road is weary that leads there?
But let the Angel take thy hand and lead thee up the misty stair,
And then with beating heart await the opening of the golden gate.

*A. A. Procter.*

Happy then are they who have already succeeded in coming home from the shipwrecks of this present life to such great joys.

O country of safety, we behold thee from afar; from this sea we greet thee, from this valley we sigh after thee; and we strive with tears, if haply we may reach thee. O Hope of mankind, Christ, God of God, our refuge and strength, whose light, beaming from afar amid the dark mists over the tempestuous sea, like the ray of a star of the sea shines brightly before us, that we may be guided to the harbour; steer our bark, O Lord, with Thine own right hand, by the rudder of Thy Cross; let us not perish in the billows: let not the raging water drown us, nor the deep swallow us up, but by the power of Thy Cross draw us from this sea to Thyself, our only consolation, Whom we can scarcely discern through our tears, from afar off, as the Morning Star and the Sun of Righteousness, awaiting us on the shore of the heavenly country.—*St. Augustine.*

All the long training and fashioning of our souls made known to us; the chastisements so wisely, so lovingly laid on us; the gentle weaning from earth; the setting free from weaknesses, failings, sins; the beginning and increases of graces; all our communions with the ever fuller imparting of the Divine Life; all the going from strength to strength until we appeared unto the God of gods in Sion. . . . Imagine all this coming back upon us, and ourselves wondering, adoring, praising God, and growing in love of Him!

Think what these words will mean in heaven: "I will call to mind Thy wonders of old time." And add to this that all around us will be a vast multitude, each one of whose memories will be filled with separate and varying histories of the miraculous Love of God.

The griefs escaped, the rocks, the shipwrecks of the soul avoided, the change from corruption to incorruption wrought out, the numberless mercies by the way, the height of glory reached. Ah! look up into the starry heavens. Marvel as more and more stars, more and more brilliant worlds come out before your gaze. Think what the heavens tell of the glory of God. And then think what the countless stars of sanctity, each star differing from the other stars in glory, will tell of His glory Who made them all to shine.—*R. M. Benson.*

